# Atrioventricular Conduction Abnormalities in Multisystem Inflammatory Syndrome in Children

**DOI:** 10.1155/2021/6124898

**Published:** 2021-10-01

**Authors:** Carlos A. Carmona, Fatma Levent, Kelvin Lee, Bhavya Trivedi

**Affiliations:** ^1^Advent Health for Children Pediatric Residency, Orlando, FL, USA; ^2^Advent Health for Children Pediatric Infectious Disease, Orlando, FL, USA; ^3^Advent Health for Children Pediatric Cardiology, Orlando, FL, USA; ^4^Advent Health Director Pediatric and Adult Congenital Electrophysiology, Orlando, FL, USA

## Abstract

Cardiac manifestations in multisystem inflammatory syndrome in children (MIS-C) can include coronary artery aneurysms, left ventricular systolic dysfunction, and electrocardiographic disturbances. We report the clinical course of three children with MIS-C while focusing on the unique considerations for managing atrioventricular conduction abnormalities. All initially had normal electrocardiograms but developed bradycardia followed by either PR prolongation or QTc elongation. Two had mild left ventricular ejection fraction dysfunction prior to developing third-degree heart block and/or a junctional escape rhythm; one had moderate left ventricular systolic dysfunction that normalized before developing a prolonged QTc. On average, our patients presented to the hospital 4 days after onset of illness. Common presenting symptoms included fevers, abdominal pain, nausea, and vomiting. Inflammatory and coagulation factors were their highest early on, and troponin peaked the highest within the first two days; meanwhile, peak brain-natriuretic peptide occurred at hospital days 3-4. The patient's lowest left ventricular ejection fraction occurred at days 5-6 of illness. Initial electrocardiograms were benign with PR intervals below 200 milliseconds (ms); however, collectively the length of time from initial symptom presentation till when electrocardiographic abnormalities began was approximately days 8-9. When comparing the timing of electrocardiogram changes with trends in c-reactive protein and brain-natriuretic peptide, it appeared that the PR and QTc elongation patterns occurred after the initial hyperinflammatory response. This goes in line with the proposed mechanism that such conduction abnormalities occur secondary to inflammation and edema of the conduction tissue as part of a widespread global myocardial injury process. Based on this syndrome being a hyperinflammatory response likely affecting conduction tissue, our group was treated with different regimens of intravenous immunoglobulin, steroids, anakinra, and/or tocilizumab. These medications were successful in treating third-degree heart block, prolonged QTc, and a junctional ectopic rhythm.

## 1. Introduction

Most pediatric SARS-CoV-2 infections are mild with 2–6% of children presenting with severe illness. Since mid-April 2020 in Europe and North America, clusters of pediatric cases with a newly described severe systemic inflammatory response and shock have appeared [1, 2]. Patients had persistent fevers >38.5°C, hypotension, features of myocardial dysfunction, coagulopathy, gastrointestinal symptoms, rash, and elevated inflammatory markers without any other obvious cause of infection. The World Health Organization, Centers for Disease Control, and Royal College of Paediatrics and Child Health associated these symptoms with SARS-CoV-2 as multisystem inflammatory syndrome in children (MIS-C) [3, 4].

Cardiac manifestations included coronary artery aneurysms in some studies describing 6–24% [2] versus 15–33% of patients within the first 2 weeks. Left ventricular systolic dysfunction is common at initial evaluation along with elevation of troponin-T (TnT) and pro-B-type natriuretic peptide (proBNP) [5]. Significant improvements in cardiac function are often seen within 30 days; however, some patients have residual low-normal function 4–6 weeks later [6]. The most often described electrocardiogram abnormalities have been non-specific ST segment/T-wave abnormalities, abnormal PR intervals, and dysrhythmias [2]. We report the clinical course of three children with MIS-C while focusing on the unique considerations for managing atrioventricular (AV) conduction abnormalities.

## 2. Patient #1

A 19-year-old previously healthy Hispanic male presented with abdominal pain, emesis, fever, body aches, and non-bloody diarrhea for three days. He was febrile and hypotensive (80/47 mmHg) requiring aggressive fluid resuscitation. Notable initial laboratory results included elevated inflammatory markers and a negative infectious workup (Table 1). Three days of fever, abdominal symptoms, hypotension, elevated inflammatory markers without a clear source of infection, and a positive COVID-19 antibody (Ab) test were consistent of MIS-C diagnosis. Methylprednisolone, intravenous immunoglobulin (IVIG), and enoxaparin prophylaxis were started in the Pediatric Intensive Care Unit (PICU). The patient was treated with epinephrine for shock and needed supplemental oxygen via high-flow nasal cannula for respiratory distress. Antibiotics including vancomycin and piperacillin-tazobactam and briefly azithromycin were used empirically.

**Figure 1:**
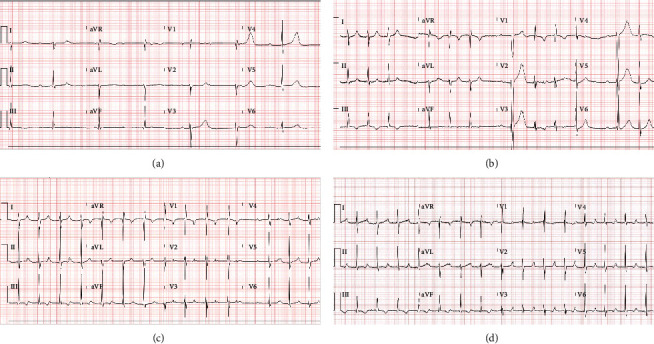
(a) Complete heart block with low ventricular rate of 41: hospital day #4—subsequently started isoproterenol. (b) Second-degree type 2 AV block: hospital day #5—on isoproterenol. (c) Second-degree Mobitz type 1 AV block: hospital day #7—on isoproterenol. (d) First-degree AV block: hospital day #8—on isoproterenol.

Initial echocardiogram demonstrated mild to moderate biventricular dysfunction with left ventricular ejection fraction (LVEF) 40% and normal appearing coronaries. Troponin was 0.41 ng/mL and proBNP was 15,301 pg/mL on admission. Electrocardiogram (ECG) showed an incomplete right bundle branch block. Approximately 12 hours from the last dose of azithromycin, the patient became bradycardic to 30s–50s; however, ECG revealed a complete AV block with a ventricular rate of 41 bpm and a junctional escape rhythm (Figure 1(a)). The LVEF had improved to 50%. Isoproterenol, a B1 cardiac receptor agonist with chronotropic activity, supported the severe bradycardia. The arrhythmia progressed to a type 2 second-degree AV block (Figure 1(b)), and the patient remained asymptomatic. A second dose of IVIG was administered. The ECG changed to a type 1 second-degree AV block (Figure 1(c)). An IL-6 inhibitor, tocilizumab, was administered. He later converted to a first-degree AV block. Isoproterenol was weaned off, but the PR interval was prolonged >250 ms, and so it was administered for another 24 hours until the condition improved. Meanwhile, anakinra was started for four days. Cardiac MRI (magnetic resonance imaging) showed septal predominant left ventricular (LV) hypertrophy and subepicardial enhancement along the basal inferior wall and mid anteroseptal walls typical for myocarditis. LVEF on echocardiogram prior to discharge home was normal, yet the patient had a PR interval of 278 ms indicative of first-degree heart block (Figure 1(d)).

## 3. Patient #2

A 9-year-old previously healthy Hispanic male presented after three days of daily fevers up to of 102 F, headaches, productive cough, diarrhea, myalgias, diffuse abdominal pain, and ageusia. He was febrile, tachycardic, and hypotensive (68/39 mmHg). Laboratory results demonstrated lactic acidosis, elevated inflammatory markers, coagulopathy, and acute kidney injury (Table 1). Infectious workup results were negative. Hypotension of 50/20 mmHg required 3 normal saline boluses of 20 ml/kg and initiation of an epinephrine drip. On arrival to the PICU, his hypoxia progressively worsened requiring endotracheal intubation. The three days of fever plus the hypotension, acute gastroenteritis, coagulopathy, signs of myocardial dysfunction without a clear source of infection, and positive COVID-19 antibody (Ab) test were diagnostic for MIS-C. Patient was treated with IVIG 2 mg/kg, methylprednisolone 1 mg/kg q 6 hrs, enoxaparin, aspirin, and ceftriaxone. On hospital day four, with persistent elevated inflammatory markers and illness severity, a 7-day course of anakinra was started. Inflammatory markers trended downwards; steroids were progressively weaned.

**Figure 2:**
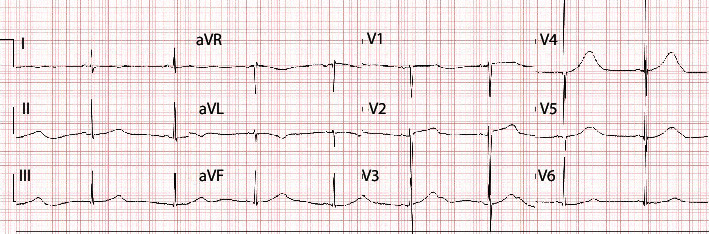
Bradycardia with prolonged QTc.

On admission, troponin was 0.33 ng/mL, BNP was 25,335 pg/mL, and ECG showed sinus tachycardia. Initial echocardiogram showed mild tricuspid and mitral regurgitation (TR/MR) with an LVEF of 35–40%. On day 2, a second echocardiogram confirmed the degree of persistent cardiac dysfunction, and so milrinone was started. After two doses of anakinra and the anti-inflammatory/anticoagulation regimen, there was resolution of mild MR/TR and LVEF normalized to 60%. He was extubated once hemodynamically stable and no longer requiring much ventilatory support. By day 7, inflammatory markers continued to trend down. Steroids were weaned to 1 mg/kg *q* 8 hrs. He was doing well clinically tolerating room air when vitals showed a trend towards bradycardia with HRs in the 50s. ECG revealed a prolonged QTc to 545 ms. He remained asymptomatic and well perfused. Bradycardia persisted into the next day as the QTc worsened to 592 ms (Figure 2). Bradycardia was simply monitored and progressively improved along with the QTc over the course of the following 3 days. He was discharged after a normal echocardiogram and QTc of 405 ms. He was discharged without complications and instructed to continue aspirin and complete the prednisolone wean.

## 4. Patient #3

A 9-year-old African American male presented with four days of right sided abdominal pain, constipation, and non-bilious non-bloody emesis. He had a negative COVID test and unremarkable ultrasound of the appendix days prior. Vitals were normal. He was coagulopathic with elevated inflammatory markers. Evaluation for infectious etiologies was negative (Table 1). The history of fevers >3 days, acute gastrointestinal illness, coagulopathy, mucocutaneous physical exam findings, elevated inflammatory markers, and a positive COVID-19 antibody test were indicative of MIS-C. IVIG 2 g/kg, prednisolone 1 mg/kg *q* 6 hrs, enoxaparin, aspirin, and ceftriaxone were started.

Initial ECG showed sinus rhythm with normal intervals and BNP was minimal. Echocardiogram showed normal biventricular systolic function. On day three, repeat showed EF of 50%. The following day, ECG revealed a right bundle branch block. Anakinra was started despite an otherwise stable clinical status. Steroids were increased to 2 mg/kg. On day five, he had asymptomatic bradycardia in the 50s shortly after methylprednisolone was decreased to 1 mg/kg. ECG progressed to a junctional rhythm, but he remained asymptomatic. Cardiac function normalized by day seven, and anakinra was stopped due to the improvements. Thereafter, HRs ranged from 38–48 bpm requiring transfer to the Pediatric Cardiac Intensive Care Unit for better monitoring and potential isoproterenol infusion. Despite the bradycardia, the patient was awake and well perfused. Heart rates improved over the next 48 hours without further intervention. Prior to discharge, ECG demonstrated sinus bradycardia with normal intervals. Echocardiogram remained normal throughout the hospital course. He was discharged home with instructions to continue aspirin and complete the prednisolone wean.

## 5. Discussion

Multiple papers have described myocardial dysfunction and the coronary manifestations associated with MIS-C [7, 8]. Non-specific T-wave, ST segment changes, and premature atrial or ventricular beats are the most often noted ECG anomalies [2, 9]. Arrhythmias have been mentioned, but few papers have discussed the evolution and progression of ECG findings to better understand its sequence in the overall course of cardiac complications. El-Assad et al. described a 10-year-old boy with transient self-resolving episodes of complete heart block with a narrow junctional escape rhythm that did not require higher intervention aside from immunosuppressants [10]. Boston Children's Hospital had a retrospective cohort of 25 MIS-C cases where AV blocks were analyzed, and it was noted that first-degree heart blocks typically progressed to high-grade AV blocks [11]. Dominco et al. described a patient with second-degree type 2 AV block with periods of an idioventricular rhythm who needed emergent transvenous bipolar pacing [12].

We report a case series of three patients with MIS-C having varying degrees of heart failure and AV conduction abnormalities. All initially had normal ECGs but developed bradycardia followed by either PR prolongation or QTc elongation. It is important to note that high-dose steroids can cause bradycardia; however, each patient received them for a different length of therapy based on downtrends of inflammatory markers. Additionally, two patients had mild LVEF dysfunction prior to developing third-degree heart block and/or a junctional escape rhythm; one had moderate LVEF dysfunction that normalized before developing a prolonged QTc. Although bradycardia may be present in many other patients being hospitalized and receiving high-dose steroids, COVID-19-induced ECG changes including heart block and bradycardia are suspected secondary to inflammation and edema of the conduction tissue as part of a widespread global myocardial injury process. Frequent ECGs were done to monitor PR prolonging/QTc elongation as they could lead to life-threatening arrhythmias requiring more aggressive cardiac support.

**Figure 3:**
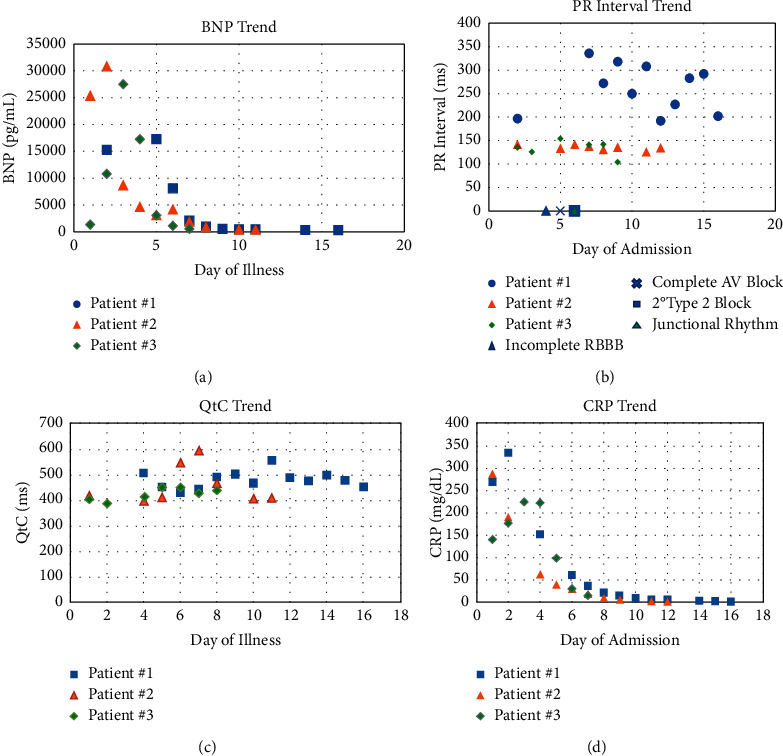
(a) Patients' BNP trend, (b) patients' PR interval trend, (c) patients' QTc trend, and (d) patients' CRP trend.

Our group on average presented to the hospital 4 days after onset of illness. Common presenting symptoms (Table 1) included fevers, abdominal pain, nausea, and vomiting. Cytokine storm was clear with elevated CRP, procalcitonin, and markers of myocardial injury with elevated troponin and BNP (Table 2). Inflammatory and coagulation factors were their highest early on while peak BNP occurred later at hospital days 3-4 (Figure 3(a)). Troponin peak was the highest within the first two days which was per the patient's lowest LVEF typically at days 5-6 of illness.

Initial ECGs were benign with PR intervals below 200 milliseconds (ms). Collectively, the length of time from initial symptom presentation till when ECG abnormalities began tended to be at days 8-9 (Figure 3(b)). Patients similarly developed increased QTc intervals later in the hospitalization (Figure 3(c)). When compared with the CRP and BNP trends, it appeared that the ECG changes (including PR and QTc elongation) occurred after the initial hyperinflammatory response (Figure 3(d)). Our results of when the initial bradyarrhythmia occurred are postpeak of inflammatory markers like El-Assaad et al. reports [10]. In addition to severe local inflammation, insufficiency of the coronary arterial supply to the atrioventricular node and specialized conduction system are the presumed mechanisms of the conduction system abnormalities [12].

The course of AV conduction abnormalities is still very unpredictable. Although our patients had normal ECG findings initially like those of Dionne et al. ours did not develop a first-degree AV block prior to higher grade ones. Two of ours developed bundle branch blocks, respectively, prior to a third-degree heart block and/or junctional rhythm. Based on the premise of this syndrome being a hyperinflammatory response likely affecting conduction tissue, our group was treated with different regimens of IVIG, steroids, anakinra, and/or tocilizumab. Anakinra, being an IL-1 inhibitor, has been reported to help with fulminant viral myocarditis in dampening inflammation and having favorable effects on cardiac contractility [13]; however, its impact of therapy and best duration of treatment are unknown. The same is true for tocilizumab.

Limitations of this retrospective case series are that further investigation is required to determine what doses of steroids can possibly cause undesired bradycardia. This includes evaluating what treatment profiles have the best efficacy and least side effects with differing severity of MIS-C symptoms. Another limitation is the interpretation of the BNP marker, which is a simple and objective measure of cardiac function and helpful to diagnose heart failure. It is primarily secreted by the ventricles as a response to left ventricular stretching or wall tension. It is activated only after a prolonged period of volume overload and helps maintain stable blood pressure and plasma volume by preventing excess salt and water retention [14]. It peaked at different times during the patients' admission. Even though it is a marker for heart failure, it needs to be assessed in the context of patients obtaining aggressive fluid resuscitation due to vasodilatory shock or receiving medication treatment like IVIG which is a high volume of fluid for some children.

## Figures and Tables

**Table 1:** Clinical features of 3 patients with multisystem inflammatory syndrome in children.

Clinical feature	Patient #1	Patient #2	Patient #3
Age/weight (kg)	19 (71 kg)	9 (31 kg)	9 (45.8 kg)
Sex	M	M	M
Race/ethnicity	Hispanic	Black, Hispanic	Black
Preexisting conditions	n/a	n/a	Obese and recent appendectomy
Acuity	PICU^€^	PICU^€^	CVICU^¥^
Co-infection	None	None	N/a
Known sources/sick contacts	None	None	Brother had complaints of abdominal pain
Abdominal pain/emesis	+	+	+
Respiratory failure	+	+	−
Shock	+	+	+
Erythematous lips	−	+	+
Headaches	−	+	−
Conjunctivitis	−	−	+

Cardiopulmonary support			
Ventilation support	HFNC^*β*^	MV^Δ^	None
Vasoactive support	Epi^ç^, Iso^£^	Epi, Mil^Ş^	None
Fluid first 24 hours	100 ml/kg	110 ml/kg	67 mL/kg

Anti-inflammatory therapies			
Intravenous immunoglobulin (2 g/kg)	100 g × 2 (2,000 mL)	68.2 g × 1 (682 mL)	85 g × 1 (850 mL)
Methylprednisolone	+	+	+
Other anti-inflammatory meds	Anakinra, tocilizumab	Anakinra	Anakinra
Antibiotics	Azithromycin, vancomycin, ceftriaxone, piperacillin-tazobactam	Vancomycin, ceftriaxone	Ceftriaxone
Initial chest radiograph findings	Right lung base opacities	Multifocal infiltrates	Normal

^€^Pediatric Intensive Care Unit. ^£^Isoproterenol. ^¥^Cardiovascular Intensive Care Unit. ^Ş^Milrinone. ^*β*^High-flow nasal cannula. ^ç^Epinephrine. ^Δ^Mechanical ventilation.

**Table 2:** Laboratory and cardiac findings.

Peak laboratory findings					

Covid PCR (nasopharyngeal)	−	−		−	
Covid AB	+	+		+	
Procalcitonin (ng/mL)	6.77	19.3		7.66	<0.10
CRP (mg/L)	333.6	285		224	<5
BNP(pg/mL)	17286	30817		27495	<450
Lactic acid (mmol/L)	2	6		2.6	0.5–1.9
IL-6 (pg/mL)	<2	−		44.8	<2.0
D-dimer (ug/mL)	3.74	14.01		6.58	0–0.5
PT/INR/PTT	17.2/1.4/30.1	19.3/1.62/46		83/109	PT: 11.5–14.9 INR: 0.8–1.2 PTT: 22.0–38.0
Fibrinogen (mg/dL)	531	502		549	188–468
Alk Phs/ALT/AST	75/163/86	186/179/82		133/138	Alk Phs: 142–335; ALT: < 51; AST: <46
CPK	683	901		1169	24–200
Ferritin	548	20814		1169	24–336
AKI (BUN/Cr)	25/1.39	70/3.3		13/0.86	Age Dependent

Cardiac findings					

Peak troponin (ng/mL)	0.41	0.33	<0.01		
Presenting ECG	Sinus tach incomplete RBBB^*π*^	Sinus tachycardia	Sinus rhythm		
Presenting PR (ms)	197	141	135		
Cardiac rhythms	Complete, first- and second-degree heart block; prolonged PR, QT	Sinus tachycardia, prolonged QT (592 ms)	Sinus arrhythmia, sinus brady, RBBB^*π*^, junctional rhythm,		
Time after symptom presentation till arrhythmia onset	Complete AV—9 days	Prolonged QT—9 days	RBBB^*π*^—8 days		
Time from onset of IVIG^Ѱ^ till arrhythmia eliminated	4	2	3		

^Π^Right bundle branch block. ^Ѱ^Intravenous immunoglobulin.
